# Comment on “Structural dynamics of cisplatin binding to histidine in a protein” [Struct. Dyn. **1**, 034701 (2014)]

**DOI:** 10.1063/1.4948613

**Published:** 2016-05-09

**Authors:** S. W. M. Tanley, J. R. Helliwell

**Affiliations:** School of Chemistry, University of Manchester, Manchester M13 9PL, United Kingdom

A re-refinement of 4mwk, namely, the X-ray triclinic crystal structure at 100 K of cisplatin binding to histidine in a protein in a multi-temperature structural dynamics study, has been made. This is so as to correct clashes in the protein structure solute molecules model for which our attention was drawn by the valuable critique of this PDB deposition by Shabalin *et al*. (2015) and that we have responded about (Tanley *et al*., 2015). The finalised model has been deposited at the PDB, to replace 4mwk. It includes the refined platinum atoms at the His15 ND and NE sites assigned as before according to their anomalous difference electron density peaks, which Shabalin *et al*. (2015) concur with. The respective platinum atoms' refined occupancies and ADPs are as in 4mwk, which were derived from SHELX least squares model refinements, as described in our article (Tanley and Helliwell, 2014). We also note that the anomalous difference densities for sulphur atoms are very clear and well resolved on the lysozyme molecule disulphides and therefore are confident on the quality of the measured anomalous difference data.

We have considered the His 15 imidazole ring split occupancy (60%/40%) placements in 4yeo but conclude that there was insufficient 2Fo-Fc electron density at the1.2 rms contour level and no Fo-Fc electron density contoured at the COOT default cut off of 5σ for such a precise interpretation. But, as we stated in our paper (Tanley and Helliwell, 2014), the His 15 imidazole ring we do agree is taking up multiple positions indicative of the structural dynamics of binding of the cisplatin, as we point out in our article.

In 4mwk, we had imported the PDB's “CPT” ligand and then deleted ligand atoms that did not fit the electron density. We realise that this was due to the PDB's CPT having an incorrect, tetrahedral, rather than square planar, geometry. In preparing this revision of 4mwk, we have now removed the CPT labels on the atoms that we had and instead assigned individual atoms and which is hopefully clearer in our PDB files.

In checking the PT labels, so as to ensure the same labelling nomenclature between the PDB files and the article, again for clarity, we noticed an error in Tables II and III concerning the carboplatin 4oxe NE side PT4 which in fact is PT1. Since 4oxe does not feature in Figure 2, being entirely cisplatin at the three temperatures, this label error in Table III does not affect Figure 2.

The revised-4mwk-cycle-27 PDB validation server report led to our deleting the top three listed poor RSR and LDF moieties, namely, “ACT N 1,” “NO3 I 4,” and “PT L.” The fourth peak DMS H 1 has a good density; we therefore retain this moiety. The fifth peak, CL J, makes a sensible ligand distance to PT C at 2.30 Å away; so we retain this ligand atom. There are some “blobs of electron density” in the COOT final Fo-Fc “validation difference map” (Emsley and Cowtan, 2004) that we have not provided an interpretation for which is because we were unsure of their chemical identities. Finally, the PDB has introduced highlighting of amino acids with “high RSRZ values”; whilst this does not apply to any of our residues we did have one ASN106 at an RSRZ of 2.9 and for which at cycle 27 there is a plausible split occupancy, and which we have accepted in our final model. Assignment of bound waters was made where there was electron density evidence for them (i.e., 2Fo-Fc contoured at 1.2 rms).

Since Tanley and Helliwell's (2014) *Structural Dynamics* article is a comparison of identically prepared crystals' diffraction data measured at three different temperatures, and carboplatin binding in the triclinic crystal form at one temperature, we have also revisited 4mwm, 4mwn, and 4oxe to again (i) remove any clashing solute molecules and (ii) replace the potentially confusing use of the incorrect “CPT.” We have removed solute molecules which we are not certain as to their chemical identities. In all these cases above (4mwk, 4mwm, 4mwn, and 4oxe), the as previously published processed structure factor amplitudes' data have been used. Table I shows that the clash scores are now much improved. The diffraction data were downloaded from the PDB for each of our deposits. We noticed a slight difference for 4mwm between the tabulated resolution 1.12 Å and that of the deposited data file 1.0 Å; in our re-refinement (5HQ1 for 4mwm), these are now consistent.

In our re-refinements above, we have harnessed the validation of the protein molecular model incorporated in the refinement program PHENIX_REFINE (Afonine *et al*., 2012). We completed the refinements with Refmac (Murshudov *et al*., 1997) for consistency. The platinum atom occupancies are those we determined by SHELX (Sheldrick, 2008).

The new PDB codes are listed in Table II along with the original PDB codes.

## Figures and Tables

**TABLE I.** The crystal structure clash scores: original and re-refined.

PDB id's	4mwk	4mwm	4mwn	4oxe
Clash scores
Original	29	17	14	12
Re-refined	2	1	3	2.5

**TABLE II.** The original PDB codes (left column) along with the new PDB codes (right column).

4mwk	5HMV
4mwm	5HQ1
4mwn	5I5Q
4oxe	5IDD

## APPENDIX: DESCRIPTION OF THE RAW DIFFRACTION IMAGES

In common with our other crystal structure studies of the platins with histidine, published in Acta Crystallographica Sections D and F, we have made available all the raw diffraction data images at the University of Manchester data repository. These raw diffraction data sets, as well as the published articles and the PDB coordinates and processed structure factors, are therefore all ***open access***. For the four crystal structures reported in our article in *Structural Dynamics*, we can now also provide the links to the respective raw diffraction data sets as follows:

(i) **4MWK_HEWL_cisplatin_Triclinic_150 K** http://dx.doi.org/10.15127/1.266899
(ii) **4MWM_HEWL_cisplatin_Triclinic_200 K** http://dx.doi.org/10.15127/1.266900
(iii) **4MWN_HEWL_cisplatin_Triclinic_RT** http://dx.doi.org/10.15127/1.266901
(iv) **4OXE_HEWL_carboplatin_Triclinic_200 K** http://dx.doi.org/10.15127/1.266902

The first four characters in each of the four lines listed above refer to the relevant, original, PDB code; for conversion to the new PDB codes, see Table I.
